# Does viewing a top-down map facilitate spatial learning from navigation in a virtual environment?

**DOI:** 10.1186/s41235-026-00732-y

**Published:** 2026-06-04

**Authors:** Jie Ding, Ho Ming Chan, Jeffrey Allen Saunders

**Affiliations:** Department of Psychology, University of Hong Kong, Hong Kong, Hong Kong

**Keywords:** Spatial learning, Navigation, Cognitive map, Map viewing, Virtual reality

## Abstract

We tested whether viewing a top-down map before or while exploring an environment can facilitate spatial learning through first-person navigation. In three experiments, participants navigated in virtual environments to find a sequence of targets (exploration) and then performed three tasks to assess their spatial knowledge: scene and orientation-dependent pointing (SOP), judgments of relative direction (JRD), and map reconstruction. In separate sessions, we tested conditions with and without map viewing. In Experiments 1 and 2, participants viewed a structural map of the environment before performing exploration trials. Experiment 1 tested city environments with rectangular intersections, and Experiment 2 tested village environments with a main path and irregular side paths. We found no evidence that the structural map previews improved overall accuracy of spatial representations learned from navigation. Experiment 3 tested a village environment with more distinctive landmarks and presented a detailed map without targets labeled repeatedly throughout the exploration and still found no benefit from viewing a top-down map for first-person navigation in large-scale virtual spaces. We speculate that difficulty in translating between egocentric and allocentric representations might make it difficult to integrate information from a map and navigation experience.

## Significance Statement

This study investigates the process of learning a cognitive map based on navigation through an environment and tests whether viewing a map can facilitate this process. Navigation is an everyday task for which people commonly use navigation aids, including maps. It is well established that people can gain spatial knowledge by viewing a map, but it is unclear whether map viewing can make it easier to learn via navigation. We tested this experimentally by presenting unlabeled top-down maps before or during navigation in virtual environments and comparing spatial knowledge to conditions with navigation alone. Surprisingly, we found no benefits from map viewing. Performance on tests of spatial knowledge was equivalent with and without map viewing even when maps were presented repeatedly during navigation. Our results suggest that it is hard to integrate information from a top-down map and direct experience when exploring a new environment.

## Introduction

With experience moving through an environment, we are able to acquire spatial knowledge about the relative locations of landmarks in addition to learning routes to destinations. Klatzky (1998) distinguishes between egocentric and allocentric spatial representations. An egocentric representation would encode the locations of landmarks relative to the observer, which would be directly applicable to guiding navigation. In contrast, an allocentric representation would encode spatial relationships in a way that is independent of the current observer location. An allocentric spatial representation is often referred to as survey knowledge or a cognitive map (O’Keefe & Nadel, 1978; Tolman, 1948). Moving through the environment provides direct information about egocentric positions of objects and landmarks, but some further processing would be required to integrate this information into a cognitive map (Burgess, 2006; Ekstrom et al., 2017; Wang, 2017).

Although we can learn the layout of a large-scale environment by navigation, the mental representation formed from navigation alone may not be accurate or consistent. People often show large errors or inconsistencies in estimates of distances within learned environments (e.g., Foo, et al., 2005; Philbeck & Learly, 2005). Map sketches after exploring an environment also tend to have large distortions in the relative locations of objects (Foo et al., 2005). Even for very familiar real-world environments, judgments of relative direction can show pointing errors as large as 40°–85° (Frankenstein et al., 2012). Extensive experience with an environment and ability to easily navigate to locations does not guarantee that people have formed an accurate representation of allocentric relationships.

Another way to acquire spatial knowledge about a large-scale environment is by viewing a map, which is qualitatively different from learning through navigation. Maps present spatial information in an allocentric reference frame, so they convey allocentric relations in an accurate and consistent way. In this study, we test whether viewing a map can facilitate the process of learning from direct navigation experience.

### Learning from map viewing vs. navigation

Some studies have compared spatial learning from studying maps and first-person navigation and found that these two learning methods had different effects on allocentric and egocentric spatial judgments (Meilinger et al., 2015; Starrett et al., 2019; Zhang et al., 2014). It is believed that spatial representation formed through direct navigation will initially be stored as route knowledge (egocentric representations), while spatial knowledge acquired through map learning will be developed into survey knowledge (allocentric representations) (Van der Kuil, 2021). Both Zhang et al. (2014) and Meilinger et al. (2015) found that spatial knowledge acquired through map viewing produced more benefit for allocentric spatial judgments, while first-person navigation contributed more toward egocentric spatial judgments. These results suggest that direct navigation experience and map learning produce distinguishable spatial representations.

### Facilitating spatial learning from navigation

In this section, we discuss how viewing a map might facilitate learning an environment through navigation.

The information from a map has properties that are complementary to egocentric representations. One limitation of egocentric representations is that distant objects and landmarks may be occluded in a single perspective view. A map can provide information about the relationship between objects and locations that are not simultaneously visible, which could help to associate information across multiple views. Another issue with learning through navigation is that object–object relations like distances and angles need to be calculated from multiple perspective views over time. Determining relative distances and angles from a map is simpler because they can be directly perceived. Thus, map information could compensate for the limitations of egocentric representations.

**Fig. 1:**
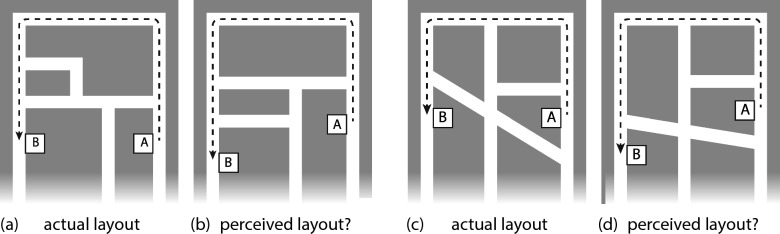
Examples of how environmental structure might be misperceived based solely on navigation experience. **a** In this environment, traveling from buildings A to B crosses one intersection on the right side and two intersections on the left side. **b** Possible misperception of the layout. Crossing multiple intersections tends to expand perceived distance, so the distance from corner to B may be perceived as longer than the distance from A to the corner. If so, it might also cause the cross streets to be associated incorrectly. **c** An example of an environment has non-orthogonal intersections, which are likely to be misperceived. **d** Possible misinterpretation if intersections are perceived as closer to orthogonal. In addition to changing the relative distances to the corners, the distortion alters the shortest path from A to B

Some of the difficulty in forming a cognitive map from navigation alone may be the inability to accurately integrate information across different views of the environment. Path integration could be used to integrate egocentric information across small distances, but accumulation of errors would limit the accuracy of path integration over longer distances (Loomis et al., 1999). If information across different views is not accurately integrated, the resulting allocentric representation could have systematic distortions or global inconsistencies. Figure 1 shows examples of how the structure of an environment and the relationship between landmarks might be misperceived based solely on navigation experience.

A map could help the integration process by providing an allocentric reference frame to organize our knowledge about landmarks and environment features. A top-down map reveals the global structure of an environment, which would otherwise have to be inferred based on a sequence of egocentric views. Over the course of navigation, information from different views could be associated with locations on the map. This could help to ensure global consistency of the allocentric representation. For the situation shown in Fig. 1a, for example, viewing a top-down map of the street layout could help an observer to correctly associate cross streets on each side of the environment, thereby avoiding the misinterpretation shown in Fig. 1b. In this way, even a minimal map could provide a useful frame for organizing information acquired during navigation.

A top-down map could also provide geometric information about the structure of an environment that supplements the information provided by an egocentric view. In the situation shown in Fig. 1c, for example, viewing a top-down map of the roads could convey the angle of the intersections better than estimating from an egocentric view. Consistent with this idea, Jansen-Osmann et al. (2007) found that presenting a structural map of a maze environment with non-orthogonal intersections prior to navigation resulted in more accurate post-test map reconstructions.

Alternatively, maps may not be very helpful for learning through navigation. If people acquire information separately from maps and navigation, the results might be stored in distinctive forms—egocentric and allocentric representations (Van der Kuil, 2021). Integrating these forms involves a translation process that requires additional cognitive resources. This might make it hard to effectively use maps to facilitate learning through navigation.

### Previous studies of map learning and navigation

Although a number of previous studies have tested spatial learning from a combination of navigation and map viewing, it is unclear whether map viewing interacts with learning through navigation.

Some studies have found that learning from combined perspectives (egocentric from direct navigation and allocentric from map viewing) produces better spatial judgments (Meilinger et al., 2015; Starrett et al., 2019). However, in these studies, it is hard to determine the source of benefit because the maps included the locations of landmarks and labeled target objects. Moreover, the map viewing conditions of these studies allowed unlimited time to view the labeled maps. It is possible that participants in these experiments learnt the spatial relationships directly from maps and did not utilize the spatial information from egocentric views in completing the spatial tasks. In both studies, the maps provided information that could be used for judgments of relative direction even without additional experience navigating in the environment. Consistent with this possibility, Starrett et al. (2019) found that spatial judgments after map viewing alone had accuracy that was comparable to combined map viewing and navigation. It is therefore difficult to determine whether viewing a map changed how information from first-person navigation was encoded, or whether effects of map viewing were due to information from the map itself.

Jansen-Osmann et al. (2007) observed a benefit from presenting a structural map before virtual exploration, rather than fully labeled maps, but their results are also inconclusive with regard to the role of the map. Jansen-Osmann et al. observed a benefit from map viewing for accuracy of map drawings but not for other measures of spatial learning. Their method of rating the accuracy of map drawings included an evaluation of the overall configuration, which could have been directly affected by viewing a structural map. From their results, it remains unclear whether viewing the structural maps facilitated learning from navigation.

Other studies have found no benefit from combining map viewing and navigation. Ishikawa et al. (2008) tested navigating with and without a global positioning system (GPS) tool and observed no difference in their spatial knowledge. In a more recent study, Yüksel et al. (2025) explored the effect of navigational aids including maps, compass, and GPS on spatial learning in large-scale virtual environments and found no difference between the groups with and without navigation aids.

In summary, previous evidence is unclear about whether spatial learning through map viewing and navigation interact, and the enhanced learning with map viewing found in some of the studies could be from direct map learning.

### Effect of maps on alignment effects

Results from Meilinger et al. (2015) provide some evidence that viewing a map can influence the reference frame for spatial knowledge gained from navigation. Judgments of relative direction typically show alignment effects (e.g., McNamara, 2002; Mou et al., 2004), which imply an orientation to the encoding of spatial layout. Meilinger et al. (2015) found the orientation of the reference frame used for spatial judgments depended on the initial experience of the environment. Participants who first learned the environment by studying a map showed alignment effects in relative judgments that were consistent with the alignment of the map, while those who first learned the environment from first-person navigation showed alignment effects consistent with their initial facing direction.

However, another study by Starrett et al. (2019) manipulated map viewing before navigation and did not find clear evidence of an effect on reference frame orientation. Starrett et al. tested learning a larger-scale environment from either navigation alone, map viewing alone, and navigation after map viewing. They found that although map viewing alone produced immediate alignment effects, map viewing before navigation did not produce an immediate alignment effect. Initial map viewing before navigation improved overall performance, but alignment effects emerged only after experience with the environment, similar to the navigation alone condition.

If map viewing before navigation influences the orientation of spatial encoding, it is possible that map viewing could influence learning in other ways. As discussed in the previous section, maps might provide an initial framework for integrating information provided by multiple egocentric views and could convey geometric information about the structure of the environment that is difficult to infer from egocentric views.

### Current study

In this study, we tested whether viewing a map of an environment interacts with learning from first-person navigation. An important difference compared to previous studies is that the maps presented in our study only showed the layout of the environment but did not include labeled locations of targets. If viewing a map enhances spatial learning through navigation by providing an allocentric frame to organize information from egocentric experience, then a minimal map showing only the general structure might be sufficient to improve cognitive maps.

**Fig. 2:**
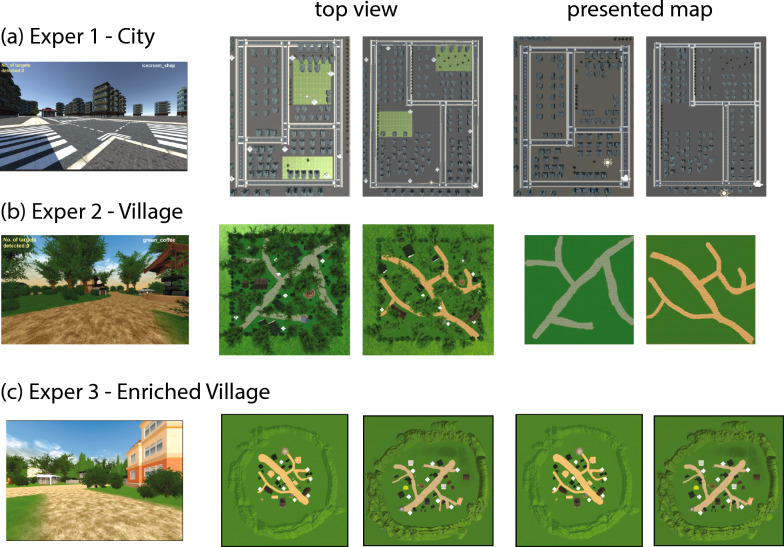
The environments used in the three experiments. The middle images show top-down views of the environments, and the right images show the maps presented in the map viewing conditions. In Experiments 1 and 2, the structural maps showed only the roads/paths. In Experiment 3, the maps showed all the features of the environment including target buildings. For the city environments, roads were aligned with the axes of the map and intersected at right angles. For the village environments, there was a main road that was diagonal on the maps, and the branching paths intersected at varied angles

The three experiments tested different types of environments and varied the degree of detail in the maps (Fig. 2). Experiment 1 used an urban environment and structural maps that showed roads. This type of environment was used in most previous studies (e.g., Meilinger et al., 2015; Zhang et al., 2014). After observing no benefit from map previews in Experiment 1, we tested a different environment. We speculated that viewing a structural map might be more beneficial for environments that do not have an underlying rectangular structure because environments with grid-like structure are generally easier to learn even without a map. Experiment 2 used a village-like environment that had a global main axis but no rectangular grid. After again observing no benefit from map previews, we made additional changes to the environment and procedure to increase the potential utility of the map viewing. For Experiment 3, we created a village environment with more unique landmarks and showed a detailed map repeatedly throughout the learning process. The maps in Experiment 3 showed the full environment including target buildings, but without target labels to prevent direct learning from the maps. The larger number of distinctive landmarks in the maps and the repeated exposure to the maps in Experiment 3 would provide more opportunity for the maps to facilitate learning from navigation.

**Fig. 3:**
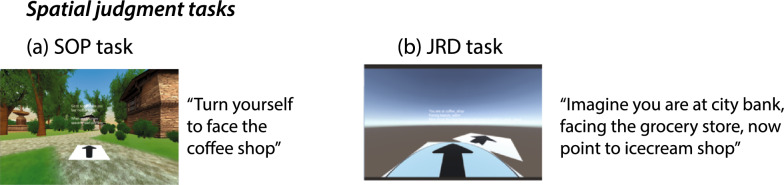
**a** The scene and orientation-dependent pointing (SOP) task. Participants were positioned in a central location in the virtual environment and asked to turn themselves to face a target that was not visible. **b** The judgments of relative direction (JRD) task. Participants were ask to imagine themselves at a target location and facing another target location and then rotate an arrow to point to a third target location

We measured spatial knowledge learned from navigation in three ways: scene and orientation-dependent pointing (SOP), judgments of relative direction (JRD), and map sketches. Figure 3 illustrates the two pointing tasks. The SOP and JRD tasks are thought to be dependent on egocentric and allocentric representations, respectively (Zhang et al., 2014). Map sketches also provide a measure of allocentric spatial knowledge.

We hypothesized that providing additional map information would improve spatial judgments that involve allocentric representations. If so, the benefit would primarily be observed in JRD performance and map sketches.

## Experiment 1—City environment

This experiment tested learning of a city-like environment with rectangular structure with and without a brief preview of the overall structure. Participants learned an environment by moving to find a sequence of target buildings. In the map preview condition, a structure map was presented at the start of each block of exploration trials. After each exploration block, participants performed the SOP task and the JRD task, in sequence, to assess their current spatial knowledge. For the JRD task, we varied the facing directions to test for map alignment effects. The environment had a natural main axis and secondary perpendicular axis. The facing directions could be aligned with the main axes or oblique directions. At the end of each session, they also performed a map reconstruction as an additional test of their allocentric knowledge.

## Methods

### Open science

This experiment was not preregistered. The data and analyses are available on the project’s OSF open archive: https://osf.io/wv3hj.

### Participants

Forty-two adults with ages 18–30 were paid $60 HKD per hour to participate in Experiment 1. The target sample size was *N* = 32. During data collection, 10 participants did not complete the experiment and were replaced. Of these 10 participants, some were stopped by the experimenter because they took more than 5 min to find all the targets in the exploration phase of the first session (*N* = 3), some withdrew due to motion sickness (*N* = 3), and others chose to discontinue for other reasons (*N* = 4). The final sample size used for analysis was 32 participants, consisting of 15 men and 17 women, with mean age of 22.7 ± 4.7 S.D. years.

We could not estimate the effect size in our experiment from previously reported results, so we chose a sample size with enough power to detect a medium-sized effect of map viewing (*d*_z_ > = 0.5). The two previous studies that tested spatial learning from navigation with and without map viewing, Meilinger et al. (2015) and Starrett et al (2019), used a between-subjects design, and the map information was qualitatively different. Converting from a between-subjects effect size to a within-subjects effect size requires knowledge of the correlation between repeated measures, which is not available. The previous studies also used a qualitatively different manipulation of map information, so would not be directly comparable. We suspected that the variability of within-subjects differences would be relatively low, so any practically relevant benefit of map viewing would have a medium or larger effect size. For these reasons, we chose to target a generic medium effect size. The sample size in our experiment needed to be a multiple of 4 for counterbalancing. With *N* = 32, the power to detect a within-subjects pairwise difference with effect size *d*_z_ = 0.5 is 78%, which is close to the typical target of 80% power. The power was calculated using the non-central t-distribution under the assumption of normally distributed noise.

Participants were required to have normal or corrected-to-normal visual acuity and were naive as to the purpose of the study. Because the virtual reality environment can often cause motion sickness, we also pre-screened for susceptibility to motion sickness and did not accept potential participants who reported that they were highly susceptible to motion sickness. All participants were compensated according to their participation time regardless of whether they completed the full experiment. The procedures were approved by and conform to the standards of the Human Research Ethics Committee for Non-Clinical Faculties.

The data were collected in 2018. Preliminary results were presented at the Annual Meeting of the Vision Sciences Society in May 2019.

### Design

We used a fully within-subjects design. All participants performed three blocks of SOP trials and JRD trials in both the map and the no map conditions. For the SOP task, the design was 2 (map/no map) × 3 (block) design. For the JRD task, alignment of the facing direction was also varied, so the design was 2 (map/no map) × 2 (aligned/oblique) × 3 (block) design. The dependent variable for the SOP and JRD tasks was the mean absolute pointing errors. Map reconstructions were performed at the end of the procedure, so there was only a comparison between map and no map conditions. For the map reconstructions, the dependent variable was the RMS errors in target positions, in normalized distance units.

### Apparatus and stimuli

Virtual displays were presented using a LED TV projector and rear-projection large screen positioned 158 cm from the observer. The image region was 158 cm wide by 84 cm tall (90° × 56° visual angle). The monocular displays were viewed with both eyes open. Participants were seated at a table and were asked to remain stationary during the experiment, but their heads were not constrained.

Two similar virtual environments were used for the two sessions for each participant. Figure 2a shows a first-person view and a top-down image of one of the two virtual environments. The environments were city scenes with streets that had right angle intersections. Buildings had variations in size and height. The environments also had features like parks and fountains to provide additional landmarks and a simulated cloud-filled sky. The overall dimensions of the environments were approximately 425 × 575 m. In each environment, eight target stores were arranged so that no more than two were visible from any location in the space. The names of the eight target kiosks were: pet care, laundry store, coffee shop, city bank, ice cream shop, beauty salon, book store, and pizza place. The kiosks had signs indicating their names that were only legible when a participant was close to the building. The environment was built using Unity 5.5.03f and some free 3D models of common objects like buildings, trees, grass fields, and street roads. The scenes were rendered using a lighting model with ambient and directional lighting.

The structural maps presented in the map preview conditions showed the layout of roads, and the main buildings with the exception of the target kiosks. The structural maps were created by printing a top-down screenshot of the Unity environment after hiding the targets and some other landmarks (Fig. 2a, right panels).

**Fig. 4:**
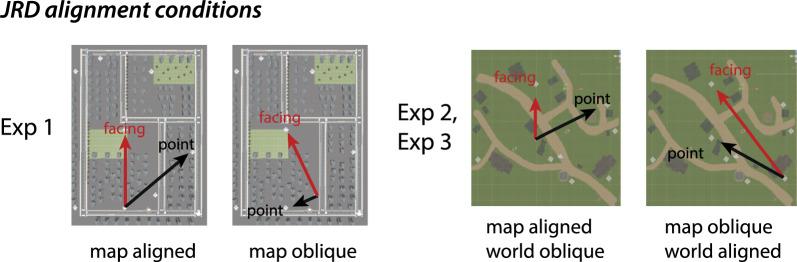
Examples of the JRD alignment conditions. The JRD combinations could be categorized into two sets based on the imagined facing direction. In the map-aligned condition, the facing direction was close to one of the main axes of the map. In the map oblique condition, the facing direction was 15-45° from any of the main axes. In Experiments 2 and 3, facing directions that were aligned relative to the map were oblique relative to the main axes of the environment and vice versa

The locations of the target stores and JRD combinations were chosen, so we could construct two alignment conditions, aligned or oblique to the main axes of the environment (Fig. 4). For trials in the aligned condition, the imagined facing direction for the JRD judgments was within 9.8° of one of the main axes of the environment, with mean deviation of 1.9°. For the trials in the oblique condition, the facing direction deviated from the nearest main axis by between 14.7° and 44.3°, with mean deviation of 26.6°.

For both scenes used in Experiment 1, the correct JRD responses were in the forward direction (i.e., within 90° of straight head) on most of the trials (11 of 12). Consequently, a biased guessing strategy could yield mean errors less than 90°. If participants always responded straight ahead, the average error would be 56.7°. Choosing a random forward direction would yield an average error of 66.8°.

### Procedure

**Fig. 5:**
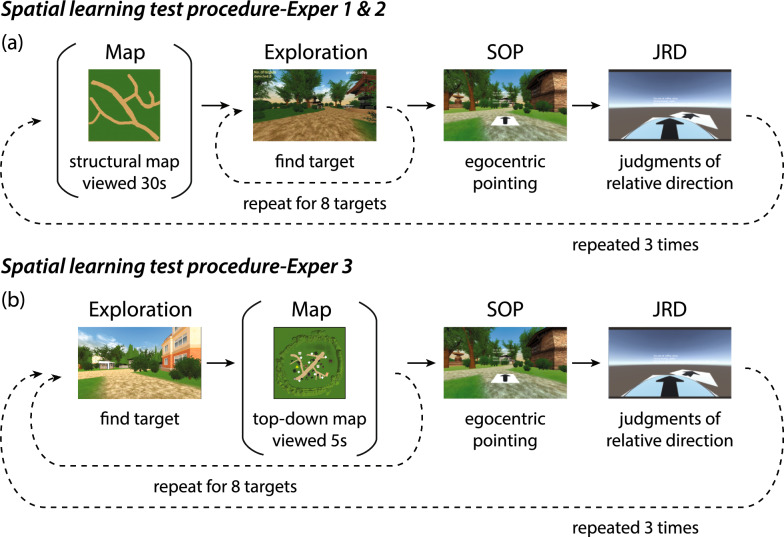
The test procedure for spatial learning and testing. Participants first learned the environment by navigating to find a sequence of 8 target buildings (exploration), and then performed SOP and JRD tasks to measure their spatial knowledge. In the map viewing condition, participants viewed a top-down structural map of the environment before exploration in Experiments 1 and 2 and a detailed map after each exploration trial in Experiment 3. The sequence was repeated three times for each condition. After the third block, participants also drew a map of the target locations on a blank A4-sized paper (not shown) in Experiments 1 and 2 and a blank paper with a circle depicting the circular boundary of space in Experiment 3

The spatial learning tasks were divided into learning and testing phases (Fig. 5), which were repeated three times for each environment. In each block, participants first explored the environment (learning phase) and then performed the SOP and JRD tasks to assess their spatial knowledge (testing phase). For all participants and conditions, the SOP task always preceded the JRD task. Map sketches were performed after the three learning and testing phases.

During the learning phase, participants actively explored the virtual environment to find a sequence of eight target stores. The name of the current target store was displayed at the top-right corner of the display. When they arrived within 0.5 m of the target, the name was updated to the next target. Forward and rotational movements were controlled with arrow keys on a keyboard. There was no time limit, but participants were encouraged to go directly to the target. We recorded the simulated positions and orientations of the observer during exploration at 10 Hz. Each learning phase ended after participants successfully located eight targets.

During the SOP testing phases, participants first navigated to a position where they felt most oriented within the scene and then judged the relative directions of a sequence of unseen targets from their position. Once participants found a position, they stayed in place for the whole block of SOP judgments. The paths and landmarks in the environment were visible during the SOP task, but the target stores were removed. The names of target stores were presented and participants rotated a virtual arrow toward the target (Fig. 3a). The task ended when a participant had pointed to all targets during a testing phase. The order of pointing targets was randomized.

During the JRD testing phases, participants were shown a blank view with only text instructions and a movable arrow. They were instructed to imagine themselves at a location, facing a specific location, and rotate the arrow so that it points to a target location (see Fig. 3b). The three locations were chosen from the set of eight target locations learned during the exploration phase. There were 12 JRD trials in each testing phase, consisting of 8 trials with aligned facing direction and 4 trials with oblique facing direction. The order of the JRD combinations was randomized in each testing phase.

The SOP task was performed before the JRD task to minimize interference between tasks. Performing the SOP is more similar to the exploration phase than the JRD. During the SOP, participants had the same view as during exploration, except for the hidden targets. Turning to face a target for the SOP task is similar to choosing a direction for initial movement on a path toward a target. For this reason, we thought that SOP would be less likely to interfere with JRD than the reverse.

In separate sessions, the participants performed the learning and testing phases with or without a map preview. In the map preview conditions, participants were given a brief view of the structure map before each exploration for 30s. The sessions with and without a preview map were performed on different days no more than seven days apart, and different environments were used in each session. We also varied the orientation of the maps. For each scene, the map could be one of two orientations differing by 90°. The order of conditions, pairing of map conditions and scenes, and map orientations were all fully counterbalanced across participants.

At the end of each session, participants were also asked to draw a map sketch showing the locations of the eight target objects. We recorded the orientation of the paper used by the participants during drawing. The drawings were scanned and the 2D coordinates of targets in page coordinates was determined using Adobe Illustrator. The sketch coordinates were normalized to compare with the correct target locations. First, the sketch coordinates were uniformly scaled so that the average distance between pairs of locations was the same as for the correct map. Both sets of coordinates were then scaled so that the maximum distance between locations in the correct map was 100. After scaling, we computed a best-fitting rotation and translation to align the drawn coordinates and the correct coordinates using the Kabsch algorithm. To reduce the effect of outliers, we applied iterative re-weighted least squares when fitting the translation and rotation, using a weighting function 1/|r|. After normalizing the drawing coordinates, we computed the mean absolute difference between the normalized coordinates and the correct coordinates and used this as a measure of the overall accuracy of the drawings. 

## Results

### JRD overall performance

**Fig. 6:**
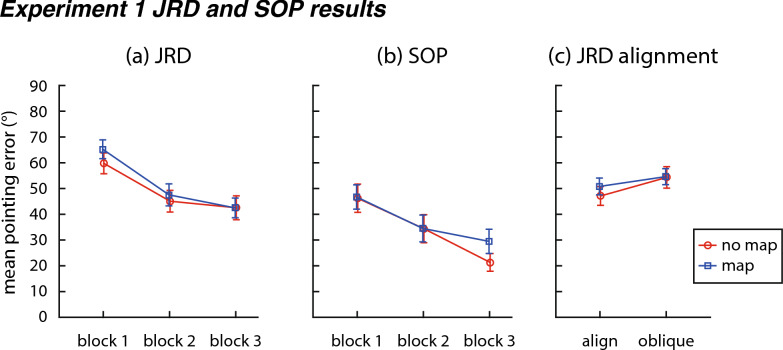
Mean pointing errors in Experiment 1. The left and middle graphs plot mean absolute angular errors from the JRD task (**a**) and the SOP task (**b**) as a function of block. The right graph (**c**) plots mean errors from the JRD task as a function of whether the facing direction was aligned or oblique relative to the map axes. The two lines on each graph correspond to the conditions with map preview (blue) and with no map preview (red)

We compared the overall JRD performance in conditions with and without a map preview, combining across alignment conditions, to test whether the map preview improved spatial learning in general. Figure 6a plots mean absolute pointing error averaged across participants as a function of learning block for the map (red) and no map (blue) conditions. In the grid environment, errors decreased across blocks, indicating that participants were learning the environment, though the errors remained fairly large (around 40 degrees) even during the final block. An 2 × 2x3 ANOVA on JRD errors found a main effect of learning block (*F*(2,62) = 22.82, *p* <.001, *η*_*p*_^*2*^ =.424), but no main effect of map preview (*F*(1,31) = 0.347, *p* =.560, *η*_*p*_^*2*^ = 0.011, *d*_z_ = 0.153), nor an interaction between map preview and block (*F*(2,62) = 0.372, *p* =.691, *η*_*p*_^*2*^ = 0.012). We also checked whether there was a difference between map preview conditions in the final block when analyzed separately and found no significant difference (*t*(31) = 0.856, *p* =.399, *d*_z_ = 0.151). There was no evidence that the map preview improved ability to make relative direction judgments, either during initial learning or after experience with the environment.

We performed an additional analysis of overall JRD performance to test whether map preview had effects on the subset of participants who performed above chance. Some participants had large average errors even after two exploration blocks, suggesting that they were either unable to learn the spatial layout or had difficulty with the JRD task. Map preview might have had more effect on the other participants. Choosing a random forward direction would yield an average error of 66.8° on our JRD trials, so we used 65° as a criteria for below chance performance. We identified 8 participants for exclusion based on average error greater than 65° in the 2nd and 3rd blocks in one of the conditions and re-ran the ANOVA on the data from the remaining 24 participants. The mean errors in the three blocks reduced to [61°, 38°, 33°], but there was no qualitative change in the pattern of results. We again found a main effect of block (*F*(2,46) = 44.8, *p* <.001, *η*_*p*_^*2*^ = 0.661), but no main effect of map preview (*F*(1,23) = 1.44, *p* =.243, *η*_*p*_^2^ = 0.059, *d*_z_ = 0.243), nor an interaction between map preview and block (*F*(2,46) = 1.02, *p* =.369, *η*_*p*_^*2*^ = 0.042). When participants performing near chance were excluded, we still found no effects of map preview on overall JRD errors.

### JRD alignment effects

For the JRD task, we further analyzed how performance varied as a function of the alignment of the facing direction relative to the map or the intrinsic structure of the environment. Previous studies have found that JRDs about locations are more accurate when the imagined facing direction is aligned with its main axes (Brunyé et al., 2015; Mou et al., 2004; Starrett et al., 2019). For the city environment in Experiment 1, this would predict better performance on trials where the participants imagine themselves aligned with the street grid than when they imagine themselves facing an oblique direction. There might also be an effect of the orientation of the map: Participants might perform better when the facing direction is aligned with the orientation of the map than when the facing direction is perpendicular, as in Meilinger et al. (2015).

Figure 6c presents the JRD performance as a function of alignment relative to the main axes of the environment. The graph plots mean JRD pointing error as a function of whether the facing direction was aligned with one of the main axes or in an oblique direction, in conditions with and without a map preview. The ANOVA results showed a significant main effect of JRD alignment (*F*(1,31) = 10.02, *p* =.003, *η*_*p*_^*2*^ = 0.244, *d*_z_ = 0.559), but no interaction between map and alignment (*F*(1,31) = 0.747, *p* =.394, *η*_*p*_^*2*^ = 0.024, *d*_z_ = 0.153) and no interaction between block and alignment (*F*(2,62) = 1.89, *p* =.159, *η*_*p*_^*2*^ = 0.058). Participants performed better overall when aligned with main axes of the environment regardless of whether they previewed the map, and there was no evidence that the alignment effect systematically changed across the three blocks.

The pattern of results was the same when participants performing near chance were excluded from the analyses of alignment effects. We repeated the analyses using the 24 participants who had mean JRD errors less than 65° in the 2nd and 3rd testing blocks of both conditions. An ANOVA on the data from these participants found a main effect of alignment condition (*F*(1, 23) = 16.315, *p* <.001, *η*_*p*_^*2*^ = 0.415, *d*_z_ = 0.825), but no significant interaction between map condition and alignment (*F*(1, 23) = 0.001, *p* =.973, *η*_*p*_^*2*^ < 0.001, *d*_z_ = 0.007) and no interaction between block and alignment (*F*(2, 46) = 1.306, *p* =.281, *η*_*p*_^*2*^ = 0.054). When participants performing near chance were excluded, we still found no evidence that the alignment effect changed with map preview or varied across blocks.

### SOP performance

We also compared the overall performance in the SOP task, which is thought to be a measure of egocentric spatial knowledge, across the map preview conditions. Due to loss of a data file, we only had complete SOP data from 31 of the participants. Figure 6b plots mean pointing errors in the SOP task as a function of testing block and with and without map preview. Overall, the errors were lower than for JRD judgments, but otherwise the pattern of results is similar. Performance improved across blocks (*F*(2,60) = 22.65, *p* <.001, *η*_*p*_^*2*^ = 0.430), but there was no main effect of map preview (*F*(1,30) = 0.418, *p* =.523, *η*_*p*_^*2*^ = 0.014, *d*_z_ = 0.116) and no interaction between map preview and learning (*F*(2,60) = 0.676, *p* =.513, *η*_*p*_^*2*^ = 0.022). There was no evidence that viewing the map improved performance of this egocentric spatial task.

The pattern of results from the SOP analysis was the same when participants performing near chance on the JRD task were excluded. We repeated the analysis of SOP errors excluding the same 8 participants who had mean error greater than 65° on the 2nd and 3rd blocks of JRD trials of both conditions. We found a main effect of block (*F* (2,44) = 22.088, *p* <.001,* η*_*p*_^*2*^ = 0.501), but no main effect of map preview (*F* (1,22) = 0.982, *p* =.333,* η*_*p*_^*2*^ = 0.043, *d*_z_ = 0.207) and no interaction between map preview and block (*F* (2,44) = 0.658, *p* =.523,* η*_*p*_^*2*^ = 0.029).

### Map sketches

**Fig. 7:**
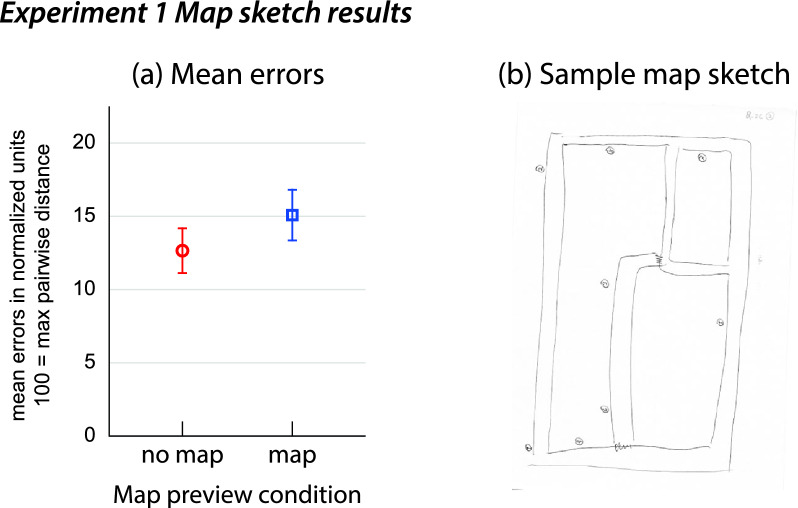
**a** The graph plots the mean errors of map sketches as a function of map preview conditions in Experiment 1. The errors are mean absolute distance errors relative to the correct map locations in normalized units, such that the maximum distance between pairs of locations is 100. The red circle shows the mean error for no map preview condition, and the blue square shows the mean error for map preview condition. Error bars depict 95% confidence intervals. **b** Sample map sketch from one participant

In addition to the JRD and SOP direction judgments, participants were also asked to draw a map of the target locations at the end of each session (see Fig. 7b for an example). We analyzed whether map preview affected the accuracy of the reconstructed map sketches.

To compute a measure of error for the map drawings, we first normalized the responses to be aligned with the correct map coordinates and then computed the mean absolute distance between drawn locations and correct locations. Participants were not constrained to draw maps at a particular scale or orientation, so the raw coordinates from map sketches would need to be scaled, rotated, and translated before they could be compared with the correct coordinates. The scaling was chosen to match the mean absolute distance between pairs of locations and to set the maximum pairwise distance in the environment to be 100 units. The rotation and translation were fit using a robust version of the Kabsch algorithm. As an error measure, we used the mean absolute distance between normalized sketch coordinates and the correct coordinates.

Figure 7a plots mean distance errors from map sketches with and without a map preview. There was no effect of map preview on the accuracy of the map sketches (*t*(30) = –1.492, *p* =.146, *d*_*z*_ = –0.268).

## Discussion

### No benefit of map preview

Experiment 1 found no evidence that viewing a structural map prior to navigating improved spatial learning. We hypothesized that a structural map might help to form an allocentric representation of an environment and thereby improve JRD judgments. However, this was not observed. For both the JRD and SOP pointing tasks, errors reduced over time, but there was no effect of map preview on either the overall performance or the rate of learning. Previewing the structural map also did not improve the accuracy of map reconstructions. These results suggest that most of the learning was from exploration, and that viewing a structural map provided little or no benefit in these experimental conditions.

Because participants performed multiple spatial judgment tasks, there might have been some interactions between tasks. The procedure was designed to minimize potential interactions. No feedback was provided for any of the tasks. The order of tasks minimized switching between an egocentric frame (exploration, SOP) and an allocentric reference frames (JRD, map reconstructions). If switching reference frames caused an interference effect, the effect on JRD performance would be similar with or without the preceding SOP task. The map reconstructions were performed at the end of a session, so could not have influenced the other tasks. However, it remains possible that the pointing tasks influenced map reconstructions, despite the lack of feedback.

We performed additional analyses to check whether the qualitative results change when excluding participants with performance near chance and found that it did not make a difference. Data from participants that had difficulty with the task could potentially have obscured a benefit from map viewing in the subset who was able to learn the layout. The results from our additional analyses suggest that this is not the case. There was no evidence for a map preview benefit even when participants performing near chance were excluded.

One possible reason that map preview had no detectable benefit is that our environment was too simple, so the map information was unnecessary. The roads were aligned with a set of perpendicular axes and had a limited number of intersections. It may have been easy to learn the overall structure just from navigating. Experiment 2 investigates this possibility by using an environment with a less regular layout of roads.

For the JRD task, the results may have had increased variability due to confusion about the task. Some participants misunderstood the instructions and made mirror-reversed responses. We improved the instructions in Experiment 2 to avoid this problem.

### Alignment effects

In Experiment 1, we observed some alignment effects in the JRD results. Performance on JRD was better when the imagined direction was aligned with axes of the city grid than when it was oblique to the axes. Similar alignment effects have been observed in previous studies (Frankenstein et al., 2012; Meilinger et al., 2015; Starrett et al., 2019).

Alignment effects were the same with and without map preview and did not vary as a function of block. This might be because the city environment had a strongly defined reference frame. The roads were aligned with the map axes and had perpendicular intersections, so there was a clear reference frame with or without a map. In the next experiment, we dissociate the salient axes of the environment from the axes of the preview map to test whether the map influences the orientation of the cognitive map.

## Experiment 2—Village environment

Experiment 2 tested the effect of map preview on spatial learning for virtual environments with a less regular arrangement of roads than in the previous experiment. Figure 2b shows a top-down view and a sample perspective view. The virtual village had a main road, but side paths were not straight and had varied angles at the intersections. The previous experiment did not find an overall benefit from map preview. This could have been because the rectangular structure made it easy to form a map-like representation even without an explicit view of a map. If this is the reason for the lack of benefit in Experiment 1, then there might be more benefit for the village environment used in Experiment 2.

We also constructed the map and environment so that the map axes were dissociated from the main axes of the environment, to test whether the preview map affected the internal orientation of the cognitive map. The main road was diagonal on the structural maps shown in the map preview conditions. The alignment effects in JRD judgments might follow either the map axes or the intrinsic axes.

An additional improvement was a modification of the JRD task to avoid confusion about the direction of response.

## Methods

### Open science

This experiment was not preregistered. The data and analyses are available on the project’s OSF open archive https://osf.io/wv3hj.

### Participants

Thirty-seven adults with ages 18–30 were paid to participate in Experiment 2. None of the participants in Experiment 2 had been tested in Experiment 1. The target sample size was *N* = 32. Five participants did not complete the experiment and were replaced. Of these 5 participants, one was stopped by the experimenter because it took more than 5 min to find all the targets in the exploration phase of the first session and the other four withdrew because of motion sickness or other reasons. The final sample size used for analysis was 32 participants, consisting of 10 men and 22 women, with mean age of 22.4 ± 3.8 S.D. years.

The results from Experiment 1 suggested that the previously used sample size (*N* = 32) would have sufficient power to detect a benefit from map viewing, so we used the same sample size in Experiment 2. Although we did not find any evidence for a map benefit in Experiment 1, some other differences were observed: an overall alignment effect in JRD accuracy (*d*_z_ = 0.56) and effect of learning across blocks (*d*_z_ > 0.9 for differences between blocks). The sample size of *N* = 32 is a multiple of 4 (for counterbalancing) and provides almost 80% power (78%) to detect an effect with size *d*_*z*_ = 0.5. If map viewing provided a benefit in Experiment 2 that was comparable to the overall alignment effect or the improvement across blocks in Experiment 1, then the sample size of *N* = 32 would provide sufficient power to detect the effect.

Participants were required to have normal or corrected-to-normal visual acuity and were naive as to the purpose of the study. Because the virtual reality environment can often cause motion sickness, we also excluded people who reported high susceptibility to motion sickness. Participants were paid $60 HKD/hour for their participation, whether or not they completed the experiment. The procedures were approved by and conform to the standards of the Human Research Ethics Committee for Non-Clinical Faculties.

The data were collected in 2019. Preliminary results were reported at the Annual Meeting of the Vision Sciences Society in May 2019.

### Design

We used a fully within-subjects design, identical to the design of Experiment 1. The design was 2 (map/no map) × 3 (block) for SOP and 2 (map/no map) × 2 (align/oblique) × 3 (block) for JRD. The dependent variable for the SOP and JRD tasks was the mean absolute pointing errors. For map reconstructions, we only compared between map and no map conditions, and the dependent variable was RMS position errors in normalized units.

### Apparatus and stimuli

In Experiment 2, the virtual environments were village scenes. Two similar scenes were used for the two sessions. In both village environments, there was a main road and branching paths, and a rectangular boundary formed by a tree wall. The main road was diagonal relative to the rectangular boundaries. Landmarks were provided by buildings, trees, grass fields, a fountain, a well, large stones, and a cloud-filled sky. The virtual villages were designed to have similar overall area and arrangement of targets as the virtual city environments used in Experiment 1. The dimensions of the environments were approximately 265 × 265 m. Figure 2b shows an example of first-person view and a top-down image of the virtual village. In each environment, eight target kiosks were arranged such that no more than two were visible at the same time. We used different names for target kiosks in the two environments to avoid confusion. In one environment, the names were: pet care, laundry store, coffee shop, city bank, ice cream shop, beauty salon, book store, and pizza place. In the other environment, the names were: village hall, country bank, pepper lunch, farmer’s market, milk house, green coffee, grocery store, and pest control.

The structural maps presented in the map preview condition showed only the layout of paths. The maps did not include the target kiosks or any of the environmental features (Fig. 2b, right). The maps were created by printing a top-down view of the Unity environment with all objects and buildings hidden.

The locations of the target stores were chosen so that we could construct two classes of JRD trials for the non-grid environment: aligned relative to the map axes, or aligned relative to the world axes. Compared to Experiment 1, the ranges of facing directions formed more distinct categories. For JRD trials aligned with the map axes, the imagined facing directions were within 5.3° of either the horizontal or vertical direction on the map, with mean deviation of 1.6°. For the JRD trials aligned with the world axis, the imagined facing directions were within 21.9° of the nearest diagonal direction on the map (at least 23.1° from horizontal or vertical) and had a mean deviation of 6.7° from the nearest diagonal (average deviation of 36.3° from horizontal or vertical). The trials with facing direction aligned relative to the world would be oblique relative to the map and vice versa (see Fig. 4 for illustration).

The correct responses on JRD trials in Experiment 2 varied over the full 360° range, so guessing would result in mean error near 90° even if a biased strategy were used. If participants always responded straight ahead, the average error on JRD trials would be 85.1°. Choosing a random forward direction would yield an average error of 87.6°.

The virtual environments were rendered and presented in the same way as in Experiment 1.

### Procedure

The tasks were the same as in Experiment 1 except for one change in the implementation of the JRD task. In the previous experiment, some participants were confused about whether the adjustable arrow indicated the direction toward the target or the direction that they were facing. This caused some participants to make mirror-reversed responses. In Experiment 2, we described the task in a more intuitive way: The fixed arrow indicates the facing direction, and the movable arrow was to be adjusted to point to the target. The results from Experiment 2 did not show any indications of mirror-reversed responses, so the new instructions appeared to have avoided confusion.

The procedure was identical to the previous experiment except for the number of JRD trials. Participants performed two sessions, with and without map preview, in counterbalanced order. Each had three repetitions of the learning and testing phase. The testing phases included both SOP and JRD judgments (with SOP always before JRD), and participants drew map sketches at the end of the last testing phase. The order of conditions and the pairing of conditions with environments were counterbalanced across participants. The only difference compared to the previous experiment is that JRD blocks had 16 trials each (48 total trials for 3 blocks), and the number of aligned and oblique trials were equal (24 trials in each condition).

## Results

### JRD overall performance

**Fig. 8:**
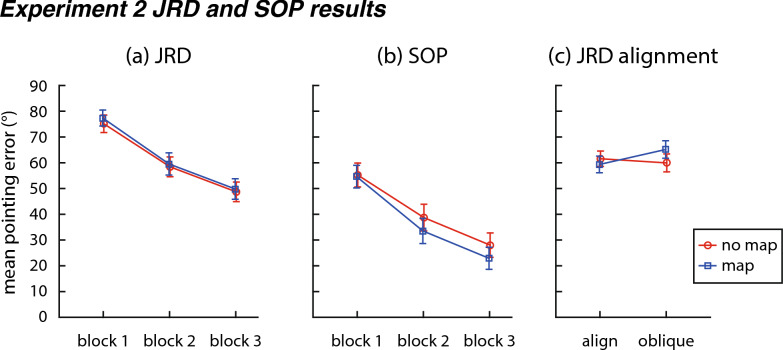
Mean pointing errors in Experiment 2. The left and middle graphs plot mean absolute angular errors from the JRD task (**a**) and the SOP task (**b**) as a function of block. The right graph (**c**) plots mean errors from the JRD task as a function of whether the facing direction was aligned or oblique relative to the map axes. The two lines on each graph correspond to the conditions with map preview (blue) and with no map preview (red)

Similar to Experiment 1, JRD performance showed evidence for learning across blocks but no evidence for a map benefit. Figure 8a plots the mean absolute pointing error, averaged across participants, as a function of learning block for the map (red) and no map (blue) conditions. Mean JRD errors dropped from about 80° in the first block to about 45° in the third block. The mean errors were larger overall than in Experiment 1. However, the errors expected from guessing are also larger in Experiment 2 (87° vs. 67°). Relative to chance performance, the mean errors are similar in magnitude across experiments.

A 2 × 2 × 3 ANOVA on JRD errors found a main effect of learning block (*F*(2,62) = 42.505, *p* <.001, *η*_*p*_^*2*^ = 0.578), but no main effect of map preview (*F*(1,31) = 0.230, *p* =.635, *η*_*p*_^*2*^ = 0.007, *d*_z_ = 0.085), nor an interaction between map preview and block (*F*(2,62) = 0.042, *p* =.959, *η*_*p*_^*2*^ = 0.001). When the final block was analyzed separately, there was also no significant difference between the conditions with and without map preview (*t*(31) = 0.284, *p* =.778, *d*_z_ = 0.050). There was no evidence that the map preview improved ability to make relative direction judgments, either during initial learning or after experience with the environment.

As in Experiment 1, we performed an additional analysis to test whether map preview had effects on the subset of participants who were able to perform above chance on the JRD task. In Experiment 2, choosing a random forward direction would result in an average JRD error of 87°, so we used 85° as a criteria for identifying below chance performance. We identified 7 participants for exclusion based on having average errors greater than 85° in the 2nd and 3rd testing blocks of one of the conditions and re-ran the ANOVA using the data from the remaining 25 participants. We again found a main effect on learning block (*F*(2,48) = 76.896, *p* <.001, *η*_*p*_^2^ = 0.762), but no main effect of map preview (*F*(1,24) = 0.035, *p* =.853, *η*_*p*_^2^ = 0.001, *d*_z_ = 0.038) nor an interaction between map preview and block (*F*(2,48) = 0.073, *p* =.930, *η*_*p*_^*2*^ = 0.003). Excluding participants who performed near chance on the JRD task reduced the mean errors in the three blocks to [76°,53°,41°], but did not change the qualitative pattern of results.

### JRD alignment effects

We further analyzed how JRD performance varied as a function of the alignment of the facing direction relative to the map or the intrinsic structure of the environment. Two categories of facing direction were tested: aligned or perpendicular relative to the upward direction of the map or aligned or perpendicular to the main axes of the world. Figure 8c plots the mean absolute pointing error as a function of alignment condition for the map and no map conditions, averaged across participants and learning blocks.

The effect of alignment in Experiment 2 was different depending on whether there was map preview. With map preview, JRD performance showed an expected alignment effect: Errors were lower for facing directions aligned with the main axis of map and higher for facing directions oblique to map axes. Without the map preview, there was a trend in the opposite direction. The ANOVA results confirmed that there was a significant interaction between map condition and JRD facing alignment (*F*(1, 31) = 6.29, *p* =.018, *η*_*p*_^*2*^ = 0.169, *d*_z_ = 0.443). Additional pairwise tests found that errors were lower for the map-aligned trials than the scene-aligned trials with when a map was previewed (*t*(31) = 2.70, *p* =.012, *d*_*z*_ = 0.474), but there was no significant difference without map preview (*t*(31) = 0.746, *p* =.461, *d*_z_ = –0.132), and the trend was in the opposite direction. The ANOVA also found that there was no main effect of alignment condition (*F*(1,31) = 1.755, *p* =.195, *η*_*p*_^*2*^ = 0.054, *d*_z_ = 0.234) and no interaction between block and alignment (*F*(2,62) = 0.456, *p* =.636, *η*_*p*_^*2*^ =.015). Although the map preview did not improve overall performance, it did change how errors depended on the alignment of the facing direction.

Excluding participants who performed near chance did not change the qualitative pattern of results from the analyses of alignment effects. We repeated the analyses using the 25 participants who had mean errors less than 85° in the 2nd and 3rd blocks. An ANOVA found a significant interaction between map condition and JRD facing alignment (*F*(1,24) = 7.542, *p* =.011, *η*_*p*_^*2*^ = 0.239, *d*_z_ = 0.549), but no main effect of alignment condition (*F*(1,24) = 1.033, *p* =.32, *η*_*p*_^*2*^ =.041, *d*_z_ = 0.203) and no interaction between alignment and block (*F*(2,48) = 0.3645, *p* =.696, *η*_*p*_^*2*^ = 0.015). When participants performing near chance were excluded, we observed the same interaction between map condition and the alignment effect and no evidence that the alignment effect varied across blocks.

### SOP performance

Similar to Experiment 1, previewing a map did not have an effect on the accuracy of egocentric pointing. We analyzed SOP mean error by learning blocks and as a function of with or without map preview. Figure 8b plots mean SOP errors as a function of block for the two map conditions. Overall performance on the SOP task improved over the three blocks, but this did not appear to be affected by the map. An ANOVA found a main effect of block (*F*(2, 62) = 34.889, *p* <.001, *η*_*p*_^*2*^ =.530), but no main effect of map preview (*F*(1, 31) = 1.194, *p* =.283, *η*_*p*_^*2*^ = 0.037; *d*_z_ = 0.193) and no interaction between map preview and block (*F*(2,62) = 0.365, *p* =.696, *η*_*p*_^*2*^ = 0.012). Thus, there was no evidence that map preview affected either the overall SOP performance or the rate of learning.

We also checked the SOP performance of participants that performed above chance on the JRD task. We re-ran the ANOVA using the SOP data from the 25 participants that had mean JRD errors less than 85° in the 2nd and 3rd blocks. We again found a main effect of block (*F*(2, 48) = 42.341, *p* <.001, *η*_*p*_^*2*^ = 0.638), but no main effect of map preview (*F*(1, 24) = 0.945, *p* =.341, *η*_*p*_^*2*^ = 0.038; *d*_z_ = 0.194) and no interaction between map preview and block (*F*(2, 48) = 0.559, *p* =.575, *η*_*p*_^*2*^ = 0.023). When participants performing near chance on the JRD task were excluded, there was no qualitative change in the results.

### Map sketches

**Fig. 9:**
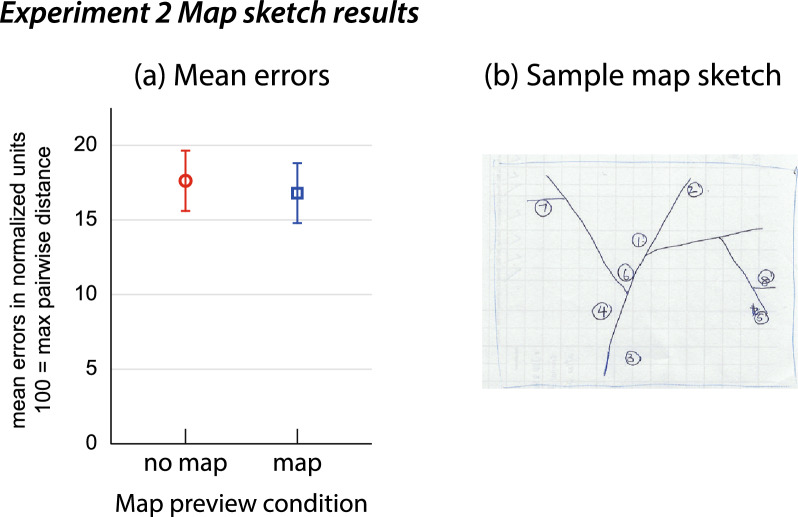
**a** The graph plots the mean errors of map sketches as a function of map preview conditions in Experiment 2. The errors are mean absolute distance errors relative to the correct map locations in normalized units, such that the maximum distance between pairs of locations is 100. The red circle shows the mean error for no map preview condition, and the blue square shows the mean error for map preview condition. Error bars depict 95% confidence intervals. **b** Sample map sketch from one participant

We analyzed map sketches in the same way as in Experiment 1. The map sketch coordinates were aligned and scaled to match the correct coordinates, with the largest distance between locations in the correct map set to be 100 units, and we computed the mean absolute distances between normalized sketch coordinates and correct coordinates. Figure 9a plots mean distance errors of map sketches with and without a map preview. There was no effect of map preview on the accuracy of the reconstructed maps (*t*(30) = 0.383, *p* =.705, *d*_z_ = 0.069).

We also checked whether the map preview affected the orientation of the map sketches. With map preview, one might expect the maps to be drawn at an orientation that is aligned with the preview map. Without the map preview, the map sketch might be more likely to be aligned with the intrinsic axes of the environment. For each participant and condition, we categorized the map sketch orientation as aligned or oblique based on whether the orientation was closer to 0°/90° axes or the 45°/135° axes. Table 1 shows the number of cases for each combination of map preview and map sketch orientation. A Chi-square test found that the orientation of map sketches was dependent on whether the map preview was shown (*X*^2^(31) = 3.97, *p* =.046). The interaction was in the expected direction: Participants were more likely to draw map sketches with an oblique main axis in conditions with a map preview.

**Table 1:** Orientation of map reconstructions in Experiment 2 with and without map preview

	Intrinsic axis drawn in oblique direction (45°/135°)	Intrinsic axis drawn in cardinal direction (0°/90°)
No map preview	*5*	*26*
With map preview	*12*	*19*

The left column shows the number of cases where the main road was drawn in an oblique direction in the reconstructed map, consistent with the preview map, and the right column shows the number of cases where the main road was drawn in a cardinal direction on the reconstructed map

It is possible that JRD alignment effects in the map preview conditions were different for subsets of participants who drew the maps with different alignments. We performed an additional exploratory analysis to test for this possibility. Participants were split into two groups according to the orientation of their map drawings in the map preview condition, and we performed a mixed 2 × 2 ANOVA on the mean JRD errors in the two alignment conditions. We found no evidence for an interaction between map sketch orientation and the JRD alignment condition (*F*(1,27) = 0.164, *p* =.689, *η*_*p*_^*2*^ = 0.006, *d* = 0.152). Note, however, that this is a between-subjects comparison with relatively small sample size, so could only detect a large difference in the alignment effects. Our data do not allow for a strong test of whether the orientation of map sketches predicts the direction of the alignment effect in the JRD task.

### Bayesian analysis of overall map benefit

Because we found no significant effects of map preview on overall performance in either experiment, we performed additional Bayesian analyses to estimate the range of possible effect sizes for the three measures of spatial learning. We compared the overall means from conditions with and without map preview after averaging across blocks and alignment conditions and combining the results from Experiments 1 and 2. For each measure, we estimated the posterior distributions for the differences between map conditions using JASP and a Cauchy prior. The results indicate that any overall benefits from map viewing in our conditions had small effect sizes. The 95% interval of credible effect sizes for a benefit from map viewing was [−0.357, 0.132] for the JRD task, [−0.228, 0.269] for the SOP task, and [−0.320, 0.177] for the map sketch task. The maximum effect sizes correspond to a 2.5° reduction in angular errors with map preview for the JRD task (4.5% reduction), a 6.0° reduction in angular errors for the SOP task (15.5% reduction), and a 12.3% reduction in map sketch errors. Although we cannot rule out the possibility that map preview had some benefits that were not detected, our results suggest that any such benefits were relatively small in our experiments.

## Discussion

### No benefit of map preview

Similar to the previous experiment, Experiment 2 did not find any evidence that map viewing facilitated learning through navigation. The three measures of spatial knowledge all showed equivalent errors with or without map preview.

Unlike the city scenes from Experiment 1, the virtual environments in Experiment 2 did not have an overall rectangular structure. We hypothesized that the rectangular structure of the city scenes might have limited the usefulness of a structural map. However, we observed similar results in Experiment 2, suggesting that the rectangular structure was not the key limiting factor.

The lack of benefit from map viewing in Experiment 2 could have been due to difficulty integrating information from the structural maps with information gained from navigation. Some participants reported that the maps were not very helpful for this reason. The structural maps showed only the intersecting paths and green grass fields. It may be hard to use this limited information, particularly when the paths do not have a rectangular structure. The irregular path structure could have also made it hard to stay oriented in the environment. Although we tried to provide distinctive features and landmarks in the environments, some participants reported that the views from different intersections looked similar. Inability to associate views with locations on the map would limit the usefulness of the map information.

### Alignment effects

The results from Experiment 2 provide evidence that the map preview influenced the orientation of the spatial representation. Performance on the JRD task showed an interaction between map preview and the imagined facing direction: With map preview, accuracy was better when the facing direction was aligned with map axes, and without map preview, accuracy tended to be better when the facing direction was aligned with intrinsic axes of the environment.

We also found that the orientation of map sketches was affected by map preview. Overall, map sketches tended to be aligned with the intrinsic axis. This tendency is stronger without a preview map and weaker with a preview map that was not aligned with the intrinsic axes. Two different measures (JRD and map sketch orientation) show that the map preview did influence the representation of spatial information, even though the map preview did not produce an overall improvement on accuracy.

Although the most salient intrinsic axis in the environments was the main paths, there were other cues that might have suggested a different orientation. The environments had rows of hedges that formed a rectangular outer boundary aligned with the map axes (Fig. 2). This rectangular structure was not obvious from a first-person view, and our results suggest that it had little influence on the way that participants oriented their cognitive maps. In the condition with no map preview, a large majority of participants (84%) drew the maps with the main path oriented vertically or horizontally, contrary to the orientation of the outer boundary.

## Experiment 3—Detailed map and immersive VR

In Experiment 3, we made multiple changes to the procedure that could facilitate use of the top-down map to improve spatial learning. One issue in the previous experiment could be the minimal information provided by the structural maps. In Experiment 3, we presented more detailed maps that included all environment features including the target buildings (which remained unlabeled). We also added more distinctive landmarks in the scene. Another possible issue in the previous experiments is the limited map exposure time. In the map viewing condition of Experiment 3, top-down maps were presented virtually after each exploration trial (see Fig. 5b). The increased map detail and more frequent exposure could potentially make it easier to use the maps to improve learning.

**Fig. 10:**
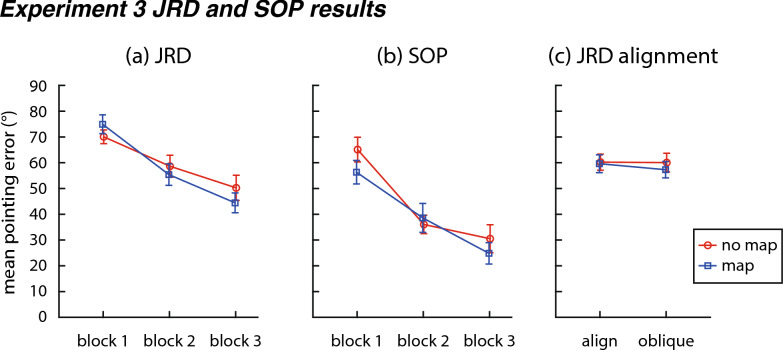
Mean pointing errors in Experiment 3. The left and middle graphs plot mean absolute angular errors from the JRD task (**a**) and the SOP task (**b**) as a function of block. The right graph (**c**) plots mean errors from the JRD task as a function of whether the facing direction was aligned or oblique relative to the map axes. The two lines on each graph correspond to the conditions with map viewing (blue) and with no map viewing (red)

To make the task more realistic, Experiment 3 used an immersive virtual reality environment with a head-mounted display (HMD) to present the virtual environment during exploration, rather than a projection screen as in the previous experiments. Using the immersive VR system, combined with the enriched virtual environments, would better simulate the experience of navigation in unfamiliar environments(Fig. 10).

## Methods

### Open science

Experiment 3 was preregistered on OSF: https://osf.io/detqh. There were no deviations from the preregistered plan. The data and analyses for Experiment 3 are available on a public OSF archive: https://osf.io/wv3hj.

### Participants

Forty-four adults with ages 18–30 were paid to participate in Experiment 3. None of the participants in Experiment 3 were tested in Experiment 1 or 2. The target sample size was *N* = 36. Eight participants did not complete experiment and were replaced. Of these 8 participants, four stopped because of motion sickness and the others chose to stop for other reasons. The final sample size used for analysis was 36 participants, consisting of 8 men and 28 women, with mean age of 24.5 ± 5 S.D. years.

Sample size was determined by a sequential procedure with possible *N* = [24,36,48,60]. Stopping was based on the JRD errors in the conditions with and without map preview. At each interim stage, we planned to perform two tests to determine stopping: a t test to detect a difference between JRD errors in the with and without map conditions and a TOST (two one-sided tests) for equivalence to determine whether the effect size of the difference is |d_z_|< 0.46. The procedure would stop with a detection if the test for differences finds *p* <.007 at *N* = 24, *p* <.012 at *N* = 36, or *p* <.02 at *N* = 48. The procedure would stop with a null result if an equivalence test for whether |d_z_|< 0.46 finds *p* <.05 (i.e., 90% CI fully contained in [–0.46, 0.46]). The combination of tests is equivalent to continuing data collection when the p value from the test for differences is within these ambiguous ranges: [.007,.60] at *N* = 24, [.012,.30] at *N* = 36, [.02,.14] at *N* = 48. At *N* = 60, data collection would stop regardless of the result and a conventional *p* <.05 will be used to determine significance. The overall false positive rate of the procedure is less than 5%. Using this procedure, data collection for Experiment 3 stopped at the second interim test with *N* = 36.

The sampling procedure was designed to target a within-subjects effect size of d_z_ = 0.46. We aimed to be able to detect a 7.5° or larger difference in the JRD errors in the map and no map conditions, which is about 15% of the expected baseline error. We estimated that the difference SD would be 16°, as found by Ding and Saunders (2019) using a similar paradigm. A mean difference of 7.5° and difference SD of 16° would correspond to an effect size d_z_ = 0.468. Our procedure has high power to detect an effect with size d_z_ > 0.46 and high power to demonstrate that |d_z_|< 0.46 if there is no difference between conditions. We performed simulations of the sequential procedure to compute expected power and sample size, with 1e7 experiments per simulation. For an effect size of d_z_ = 0.46, the procedure has 90% power to detect an effect using an average sample size of *N* = 38.3. For a smaller effect size of d_z_ =.40, the procedure has 80.9% power to detect an effect using an average sample size of *N* = 40.6. If there was no difference between conditions, the false positive rate is less than 5% and average sample size is *N* = 35.1. In the null case, we also evaluated the power for a TOST equivalence test of whether |d_z_|< 0.46. If there is no difference between conditions, there is a 94.4% probability that the confidence interval for effect size will be fully contained within [-.46,.46].

Participants were required to have normal or corrected-to-normal visual acuity and were naive as to the purpose of the study. Because the virtual reality environment can often cause motion sickness, we also excluded people who reported high susceptibility to motion sickness. Participants were paid $60 HKD/hour for their participation, whether or not they completed the experiment. The procedures were approved by and conform to the standards of the Human Research Ethics Committee for Non-Clinical Faculties at The University of Hong Kong. The data were collected in 2023.

### Design

We used a fully within-subjects design, identical to the design of Experiments 1 and 2. The design was 2 (map/no map) × 3 (block) for SOP and 2 (map/no map) × 2 (map align/oblique) × 3 (block) for JRD. The dependent variable for the SOP and JRD tasks was the mean absolute pointing errors. For map reconstructions, we only compared between map and no map conditions, and the dependent variable was RMS position errors in normalized units.

### Apparatus and stimuli

Experiment 3 used an immersive virtual reality system to run the learning and testing phases. We used an HTC Vive system with a head-mounted display (HMD) and a handheld controller to present the virtual environments. The HMD had a 110° field of view with a total resolution of 2160 × 1200 pixels (1080 × 1200 pixels per eye) and a 90 Hz refresh rate. A laptop computer with a NVIDIA GeForce GTX970 graphic card was used to render the virtual environment and administer the JRD task.

Virtual movement was a combination of simulated translation and physical rotation. The touchpad of the handheld controller was used to control translation. The maximum translation speed was 3.5 m/s. Rotations in the virtual environment were performed by physically turning, which reduced the potential for motion sickness.

Unlike the previous experiments that presented structural maps, the maps shown in Experiment 3 were top-down images of the full environment. The maps were created by taking a top-down view of the Unity virtual environments and were displayed with the virtual head-mounted displays.

Two new virtual environments were constructed for Experiment 3. The environments were village environments, as in Experiment 2, but with a larger number of distinctive landmarks. The environments had a main path and several branching paths. The main road was diagonal relative to the maps. Landmarks were provided by distinctive buildings, trees, grass fields, a fountain, a well, large stones, and a cloud-filled sky. The boundaries of the environment were circular in Experiment 3 to avoid presenting a reference frame that conflicts with the orientation of the main road. The virtual villages were designed to have similar overall area and arrangement of targets as the virtual environments used in Experiments 1 and 2. The dimensions of the environments were approximately 213 × 213 m. In each environment, eight target kiosks were arranged such that no more than two were visible at the same time. We used different names for target kiosks in the two environments to avoid confusion. In one environment, the names were: pet care, laundry store, coffee shop, city bank, ice cream shop, beauty salon, book store, and pizza place. In the other environment, the names were: village hall, country bank, pepper lunch, farmer’s market, milk house, green coffee, grocery store, and pest control.

The locations of the target stores were chosen so that we could construct two classes of JRD trials for the enriched village environment similar to previous experiments: aligned relative to the map axes or misaligned (aligned with the intrinsic axes). The locations and combinations for JRD trials were approximately the same as in Experiment 2.

For the map sketching task, we presented participants with a sheet of paper with a large circle that represents the circular boundary of the environment. In the previous experiments, participants made their map sketches on blank papers. The additional circle was added in Experiment 3 to provide a size references for the map sketches.

### Procedure

The task procedures were the same as in Experiment 2 except that the top-down maps were shown repeated within VR. In the map viewing conditions of Experiment 3, the top-down map was presented for 5s after each exploration trial (Fig. 5b).

Participants performed two sessions, with and without top-down map viewing, in counterbalanced order. Each had three repetitions of the learning and testing phase. The testing phases included both SOP and JRD judgments (with SOP always before JRD), and participants drew map sketches at the end of the last testing phase. The order of conditions and the pairing of conditions with environments were counterbalanced across participants. In Experiment 3, JRD blocks had 12 trials each (36 total trials for 3 blocks), and the number of aligned and oblique trials was equal (18 trials in each condition).

## Results

### JRD overall performance

Similar to Experiments 1 and 2, JRD performance showed evidence for learning across blocks but no evidence for a map benefit. Figure 10a plots the mean absolute pointing error, averaged across participants, as a function of learning block for the map (blue) and no map (red) conditions. Mean JRD errors dropped from about 75° in the first block to about 45° in the third block. The mean errors are smaller than in Experiments 1 and 2. Relative to chance performance, the mean errors are similar in magnitude across experiments.

A 2 × 2 × 3 ANOVA on JRD errors found a main effect of learning block (*F*(2,70) = 32.998, *p* <.001, *η*_*p*_^*2*^ = 0.485), but no main effect of map viewing (*F*(1,35) = 0.134, *p* =.717, *η*_*p*_^*2*^ = 0.004) nor an interaction between map viewing and block (*F*(2,70) = 2.922, *p* =.060, *η*_*p*_^*2*^ = 0.077). When the final block was analyzed separately, there was also no significant difference between the with and without map viewing conditions (*t*(35) = 1.3, *p* =.203, *d*_z_ = 0.216). There was no evidence that having a detailed top-down map view after each exploration trial improved the ability to make relative direction judgments, either during initial learning or after experience with the environment.

### JRD alignment effects

We further analyzed how JRD performance varied as a function of the alignment of the facing direction relative to the map or the intrinsic structure of the environment. Two categories of facing direction were tested: aligned or perpendicular relative to the upward direction of the map or aligned or perpendicular to the main axes of the world. Figure 10c plots the mean absolute pointing error as a function of alignment condition for the map and no map conditions, averaged across participants and learning blocks.

Unlike the previous experiment, we did not observe an alignment effect that depended on map viewing. An 2 × 2 ANOVA on map conditions and JRD alignment, averaging across blocks, found no significant interaction between map condition and alignment (F(1,35) = 0.417, *p* =.523, *η*_*p*_^*2*^ = 0.012). There was also no main effect of either map condition(F(1,35) = 0.213, *p* =.647, *η*_*p*_^*2*^ = 0.006) or alignment (F(1,35) = 0.717, *p* =.403, *η*_*p*_^*2*^ = 0.020).

### SOP performance

Similar to Experiments 1 and 2, having a view of detailed map after each exploration trial did not have an effect on the accuracy of egocentric pointing. We analyzed SOP mean error by learning blocks and as a function of with or without map viewing. Figure 10b plots mean SOP errors as a function of block for the two map conditions. Overall performance on the SOP task improved over the three blocks, but this did not appear to be affected by the map. An 2 × 3 ANOVA found a main effect of block (*F*(2, 70) = 33.284, *p* <.001, *η*_*p*_^*2*^ = 0.487), but no main effect of map viewing (*F*(1, 35) = 0.723, *p* =.401, *η*_*p*_^*2*^ = 0.020) and no interaction between map viewing and block (*F*(2,70) = 1.954, *p* =.149, *η*_*p*_^*2*^ = 0.053). Thus, there was no evidence that detailed top-down map viewing affected either the overall SOP performance or the rate of learning.

### Map sketches

**Fig. 11:**
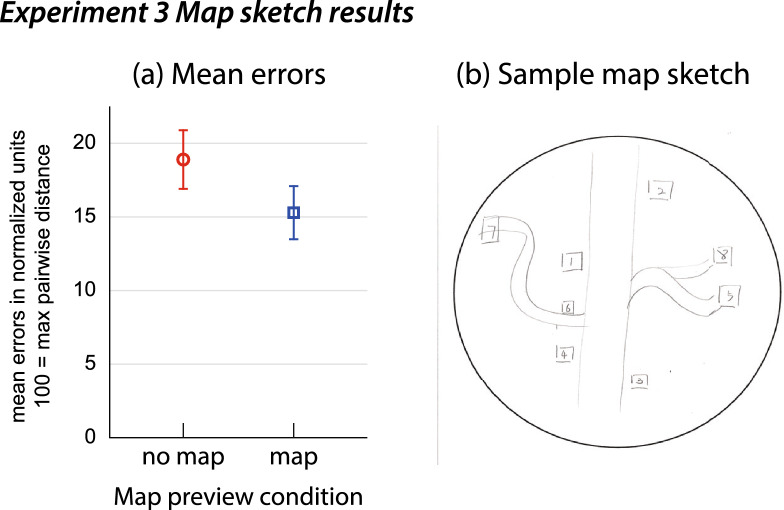
**a** The graph plots the mean errors of map sketches as a function of map preview conditions in Experiment 3. The errors are mean absolute distance errors relative to the correct map locations in normalized units, such that the maximum distance between pairs of locations is 100. The red circle shows the mean error for no map preview condition, and the blue square shows the mean error for map preview condition. Error bars depict 95% confidence intervals. **b** Sample map sketch from one participant

We analyzed the map sketches in the same way as in the previous experiments. Figure 11a plots mean distance errors of map sketches with and without map viewing. There was no effect of map preview on the accuracy of the reconstructed maps (*t*(35) = 1.901, *p* =.066, *d*_z_ = 0.317). An example map sketch from a participant in Experiment 3 is shown in Fig. 11b. In this example, the upward direction of the reconstructed map is aligned with the intrinsic axes of the world.

We also checked whether the map preview affected the orientation of the map sketches. For each participant and condition, we categorized the map sketch orientation as aligned or oblique based on whether the orientation was closer to 0°/90° axes or the 45°/135° axes. Table 2 shows the number of cases for each combination of map preview and map sketch orientation. A Chi-square test found that the orientation of map sketches was not dependent on whether the detailed map was shown (*X*^2^(35) = 1.532, *p* =.215).

**Table 2:** Orientation of map reconstructions in Experiment 3 with and without detailed map viewing

	Intrinsic axis drawn in oblique direction (45°/135°)	Intrinsic axis drawn in cardinal direction (0°/90°)
No map viewing	*10*	*26*
With map viewing	*15*	*21*

The left column shows the number of cases where the main road was drawn in an oblique direction on the reconstructed map, consistent with the preview map orientation, and the right column shows the number of cases where the main road was drawn in a cardinal direction on reconstructed map

It is possible that JRD alignment effects in the map viewing conditions were different for subsets of participants who drew the maps with different alignments. We performed an additional exploratory analysis to test for this possibility. Participants were split into two groups according to the orientation of their map drawings in the map preview condition, and we performed a mixed 2 × 2 ANOVA on the mean JRD errors from trials in the map viewing conditions with aligned and oblique JRD orientations. We found no evidence for an interaction between map sketch orientation and the JRD alignment condition (*F*(1,34) = 0.989, *p* =.327, *η*_*p*_^*2*^ = 0.028).

### Bayesian analysis of overall map benefit

We performed additional Bayesian analyses to estimate the range of possible effect sizes for the three measures of spatial learning for Experiment 3. Similar to Bayes analysis for Experiments 1 and 2, we compared the overall means from conditions with and without map preview after averaging across blocks and alignment conditions. For each measure, we estimated the posterior distributions for the differences between map and no map conditions. For the JRD task, the 95% interval of credible effect sizes for a benefit from map viewing was [−0.266, 0.388]. This indicates that any overall benefit from map viewing for the JRD task was a small effect. For the other tasks, the results do not rule out a medium-to-large effect of map viewing. The 95% interval of credible effect sizes was [−0.184, 0.467] for the SOP task and [−0.018, 0.656] for the map sketch task. Although we did not detect significant benefits from map viewing, the results do not rule out a non-trivial effect, especially for the map sketch task.

## Discussion

Despite making changes to the method that could have helped participants to utilize the map information, Experiment 3 found no evidence that map viewing during navigation improved spatial learning.

The results provide evidence against some possible explanations for the null results in the previous experiments. For Experiments 1 and 2, we hypothesized that the null result of map viewing might have been due to limited exposure to the maps and the lack of distinctive features in the preview maps. The design and procedure of Experiment 3 improved both of these factors. The maps included more distinctive features and were presented many times throughout the learning process. The lack of benefit from map viewing in Experiment 3 suggests that these were not the key factors that contributed to the null results in the previous experiments.

We did not replicate the JRD alignment findings of Experiment 2. The previous experiment found that map preview could influence the orientation of spatial representations. With map preview, more participants in Experiment 2 (dissociable map and intrinsic axes) performed better in the spatial judgment task when aligned with the map axes. However, in Experiment 3, there was no interaction between map viewing and the JRD alignment effect nor any overall alignment effects. Experiment 3 also did not find any difference in the orientation of map sketches.

The discrepancy between Experiments 2 and 3 might be due to the order of presenting navigation and maps. A previous study by Meilinger et al. (2015) found that alignment effects depended on whether participants first experienced navigation or map viewing. In our Experiment 2, the preview map was shown before any navigation, and the JRD and map sketch results indicated a tendency to adopt the reference frame of the map rather than the intrinsic reference frame. In our Experiment 3, the first presentation of the map was after completing the first navigation trial, and we found no JRD alignment effects and map sketches tended to be aligning with intrinsic reference frame regardless of map viewing conditions.

## General discussion

### No detectable benefit from map viewing

We found that brief viewing of a structural map before or after navigating in an environment did not produce measurable improvement in spatial knowledge. The accuracy of JRD judgments was similar with and without map viewing, and there was no difference in the rate of learning. Consistent findings were observed both for a city grid environment (Experiment 1) and a less structured village environment (Experiment 2) with map preview and for a village environment with repeated map viewing throughout exploration (Experiment 3). The results from our Bayesian analyses of combined data indicate that if there were effects of map viewing that were not detected, the amount of improvement was small for all of our measures.

The lack of benefit from map viewing appears inconsistent with results of some previous experiments that reported benefits. Meilinger et al. (2015) found that spatial judgments were better for participants who studied a map before navigation than those who did navigation prior to map reading, suggesting that the map facilitated learning. However, the improvement was only observed for some orientations, so could reflect an alignment effect. Starrett et al. (2019) found that map viewing before navigation improved accuracy compared to navigation alone, though this benefit reduced with practice. In contrast, our results provided no evidence for benefit from map viewing either early or late in the learning process.

One important difference is that the previous studies showed a full labeled map, so participants could have learned the environment directly from map viewing in the previous studies. Because the maps in previous studies included the names and locations of the targets, participants could make judgments about the spatial layout even with no navigation experience. Starrett et al. (2019) observed that map learning alone resulted in smaller initial pointing errors compared to map viewing before navigation, demonstrating that participants were capable of using the map information directly. In our study, the maps could potentially help people integrate what was learned from navigation, but participants could not perform the spatial judgment tasks solely from the maps. A possible explanation for the contrasting findings is that people are able to use fully labeled maps to learn a layout, as shown in previous studies, but cannot easily use structural maps or unlabeled maps to improve learning through navigation, as found in our study.

The null findings in these experiments are consistent with a previous study from our laboratory that found no benefit from providing vista views during navigation (Chan et al., 2023). In Chan et al. (2023), we presented un-occluded views of the environment during exploration, which is similar to showing a large unlabeled map in perspective view. As in the present study, Chan et al. (2023) found no evidence that the additional information improved spatial learning through navigation.

### Alignment effects

We did not observe consistent alignment effects across experiments. In Experiment 1, which tested rectangular environments, JRD responses showed an overall alignment effect favoring facing directions that were aligned with the city grid. Experiment 2 observed alignment effects that depended on whether a map was viewed, and Experiment 3 found no alignment effects at all.

The results from Experiment 2 suggested that map viewing might influence how spatial orientation gets encoded, which would be consistent with previous findings by McNamara (2002) and Mou et al. (2004). When intrinsic and map axes are aligned, in Experiment 1, participants performed better when imagining themselves aligned with the horizontal and vertical directions regardless of map condition. When the environmental axis and the map axes were dissociated, in Experiment 2, participants tended to perform better when aligned with map axes in the conditions with map viewing but better when aligned with environment axes without map viewing.

However, Experiment 3 did not replicate the interaction between map viewing and alignment effects that was observed in Experiment 2, nor any overall alignment effect. The interaction in Experiment 2 was not very large, so it is possible that it is not a reliable effect or depends on other factors. Another possibility is that the discrepancy between Experiments 2 and 3 was due to order of presentation (see Experiment 3 discussion). Previous evidence by Meilinger et al. (2015) suggested that spatial representations are formed by the first exposure. When participants first viewed the environment in first person (Experiment 3), the spatial representation are oriented aligning with the first-person perspective (more people drew map sketches aligned with intrinsic axes), whereas when first exposure was with a top-down view (Experiment 2), the spatial representations were more likely to be map aligned.

### Why no benefit from map viewing?

Even if maps are unlabeled they provide structural information that could potentially facilitate spatial learning from navigation. Why did we observe no evidence for a benefit?

One limitation in Experiments 1 and 2 is that the structural maps had limited information. Originally, we thought providing structural information like intersections and boundaries of the streets could serve as geometric anchors to help organize spatial information gained from navigation. However, no benefit was observed. It is possible that additional salient landmarks are needed to associate maps with first-person views. Therefore, we added unlabeled landmarks in Experiment 3 together with the structural layouts to test if adding additional distinctive landmarks could help spatial integration from multiple views.

Another possible limitation of our experiments is the time of map exposure. In Experiments 1 and 2, we only allowed for 30s map preview before each block of trials, so total exposure time was 90s. In Experiment 3, participants repeatedly viewed a map 24 times for 5s per trial, corresponding to a total exposure time of 120s. The amount of exposure to maps might not be enough to effectively use the map information. On the other hand, the exposure times we used are comparable to those of previous experiments of map learning (Dedetaş Şatır et al., 2026; Jansen-Osmann et al., 2007; Meilinger et al., 2015), and the pattern of repeated short exposures in Experiment 3 is an approximation of how people might refer to maps in real-world navigation. We believe that our experiments provide a reasonable amount of map exposure, but it remains possible that longer exposures could have allowed a map benefit to emerge.

It is possible that participants did not have enough experience with the environment to effectively use the map information. People might need some level of allocentric spatial knowledge before they can associate map information with navigation experience. Some researchers have argued that formation of survey knowledge through navigation requires repetitive exposure (Montello et al., 2004; Siegel & White, 1975). If participants in our experiment did not have sufficient experience navigating the environments to form allocentric representations, it may have prevented them from using information from the 30s one time preview map and 5s brief map viewing between exploration trials. With more exposure through navigation, participants might have been able to utilize map information in organizing spatial relationships and improve overall task performance.

The limited benefit could be from a general difficulty in associating map knowledge with direct experience during navigation. Integrating these sources of information requires translating between egocentric and allocentric coordinates. There is evidence that overlapping yet distinctive brain regions are involved with egocentric and allocentric encoding of spatial information (Zhang et al., 2014). Egocentric spatial representations are found mostly in regions upstream of the hippocampal–entorhinal allocentric representations (e.g., primary sensory cortex), with some overlaps (e.g. in the hippocampus and lateral entorhinal cortex) (Wang et al., 2020). The differential brain networks engaged for the two spatial representations suggest distinctive but overlapping encoding. Even if people were able to develop two frames of references in parallel, there may be a translation cost (Meilinger et al., 2015; Shelton & McNamara, 2004). Such switching cost has been commonly found in visual perspective taking tasks where change of view is involved (Segal, 2025). Some researchers have proposed that the mental representation of allocentric spatial information formed through direct experience is not map-like (Ekstrom et al., 2017; Wang, 2012; Warren et al., 2017). If the allocentric spatial representations are not map-like, it may be hard to translate between these representations and information from a map. This might explain why participants in our experiment were not able to effectively use the map information to improve their spatial judgments.

The ability to use maps to facilitate spatial learning might vary across individuals depending on visual–spatial ability. People who can readily associate map information with egocentric views might derive more benefit from combining map viewing with navigation (Ishikawa & Montello, 2006; Van der Ham et al., 2020). Evidence from an indoor navigation study using real 2D and 3D maps showed that individuals with higher self-reported Santa Barbara Sense of Direction score (SBSOD) make better use of simple maps than those with lower scores (Stites et al., 2020). Although our sample sizes were reasonable for group-level comparisons, they are not enough to test for such individual differences. In addition, our participants were relatively homogeneous (college students). Further research with a larger and more diverse sample might reveal that map viewing during navigation is more beneficial for some subsets of people in map utilization.

## Conclusion

We tested whether briefly viewing a top-down map before or during exploratory navigation could facilitate learning and observed no overall benefit. We found some evidence that previewing a structural map before navigating could influence the orientation of the mental representation of space (Experiment 2), but otherwise we found no difference between performance with and without maps across multiple tasks. We speculate that difficulty in translating between egocentric and allocentric representations may have prevented participants from effectively using map information.

## Open science statement

Experiment 3 was preregistered on OSF: osf.io/detqh. Experiments 1 and 2 were not pre-registered. All data reported here are available in a public OSF repository: https://osf.io/wv3hj.
